# Some Account of a Swine Pestilence
*The North American Medico-Chirurgical Review. May 1858.


**Published:** 1858-10

**Authors:** 


### SOME ACCOUNT OF A SWINE PESTILENCE.*

We have often endeavoured to fix the attention of the readers of this Review, on the importance of studying the relationships which may exist between the epizootic and the epidemic diseases. We have shown that man is sometimes connected by disease, as by a common tie, with his lower earth-mates. We have shown that through the inferior animal, the higher animal may receive certain of his maladies, and the converse; and we have tried to point out, that in a correct study of those epizootic diseases which are communicable, there is opened the widest sphere of experimental research, and is laid the soundest foundation for the construction of the positive of science, in matters relating to the laws and evidence of contagion.

In pursuance of our previous observations, we this time offer some account of a remarkable epizootic amongst swine, in the United States of America. We had heard of the disease incidentally, at our last issue, but not with sufficient accuracy of detail to warrant any description. This quarter, we are more fortunate. The *North American Medico-Chirurgical Review* for May, contains an able article on the subject, from the pen of Dr. George Sutton, of Aurora, Dearborn County, Indiana. Dr. Sutton has made a long series of researches on the epizootic, and has contributed a paper which will not soon be lost in the rolls of scientific history. From this paper we shall borrow in full all the information it affords, as to the origin, nature, and transmission of the new disease visitor.

The epizootic we are about to describe has received a name. The lay public have called it the "*Hog-Cholera*". The term was chosen hastily, and rather as expressing the virulence and the mortality of the disorder, than the symptoms by which it is characterised. The vast territories over which the pest has swept may be conceived, when the fact is stated, that it has prevailed in the states of Illinois, Kentucky, Indiana, Ohio, New York, Massachusetts, Pennsylvania, and Maryland; spreading widely among the swine family whithersoever it went, and destroying so wholesale, that one distiller alone, in Indiana, lost fourteen hundred hogs in one month, September.

Dr. Sutton very graphically depicts the malady. There was a premonitory stage, during which the hog appeared weak; his head drooped, and sometimes, in a few hours after these

* The North American Medico-Chirurgical Review. May 1858.

symptoms, diarrhœa commenced. There was frequently vomiting. In some cases, the discharges were serous and clay-coloured; sometimes they were dark, sometimes they were muco-sanguineous, resembling the excreta of dysentery. The urine, at first, was generally small in quantity and high-coloured; but as the animal recovered it became abundant and clear; by this sign the men who attended on the animals knew that convalescence was probable. In a large number of cases the respiratory organs seemed to be the parts most affected; there was coughing, wheezing, and difficult respiration. In some instances the animal lost the power of squealing, and the larynx was diseased. There was frequently swelling of the tongue, and bleeding from the nose. In those cases where the respiratory organs were the principal seat of the disease, there was generally no diarrhœa or dysentery. In many instances the disease appeared to be principally confined to the skin; sometimes the nose, the ear, or the side of the head, were very much inflamed; the ear being swollen to twice its usual thickness. The inflammation would spread along the skin, sometimes over the eye, producing complete blindness. Occasionally one or more legs were inflamed or swollen, and the inflammation also extended along the body. The skin, where it was inflamed, was red and swollen. Some had large sores on their flanks, or sides, from three to six inches in diameter. In one instance, at the distillery, the inflammation extended along the fore leg, the foot became ulcerated and sloughed off, and the animal recovered. Some appeared delirious, as if there was inflammation of the brain. Dr. Sutton examined the blood of four hogs which had this disease well marked; they were destroyed by the knife, and the blood, arterial and venous, was caught in a bowl. It was cupped, and presented a well marked buffy coat. Death took place in from one to ten days after the attack. Sudden changes in the weather, particularly from warm to cold, appeared to increase the fatality of this disease. The average mortality of hogs that were in pastures or fed on slop, was from thirty-three to forty-five per cent.; but it was frequently much more fatal if hogs were fed on corn, in some instances ranging from seventy to eighty out of the hundred, and, in many instances, even higher.

From the description of the symptoms, Dr. Sutton describes with great exactness the pathology of the disorder. He gives in this part, his experience of the pathology as it was derived from sixty-seven *post mortem* examinations.

In all cases the inflammation showed evidences of its diffusive character, not being confined in any instance to any one particular tissue. The skin frequently presented patches of inflammation, and often had a purple appearance. In cutting through parts that were the most inflamed, the skin was swollen, and the cellular tissue was infiltrated with serum. Frequently, however, the skin was merely discoloured, without any swelling whatever. The stomach was occasionally distended with food, and the mucous membrane in nearly every instance presented evidence of inflammation, sometimes extending over the whole stomach, at others, only in patches; it was generally of a deep red colour, thickened, and frequently softened. Sometimes it was covered with a viscid mucus, in other instances there was an effusion of blood into the stomach. The mucous membrane of the small or large intestines, where there had been diarrhœa or dysentery, presented in all instances evidences of inflammation; in patches it was red, thickened, sometimes softened, and occasionally ulcerated; where there had been dysentery, there was generally bloody mucus found in the large intestines. The bladder generally contained urine; sometimes its mucous membrane was inflamed, and in one instance there was an effusion of blood into this organ. In a large number of cases, Dr. Sutton found evidences of peritoneal inflammation, such as redness of this membrane, effusion of turbid or bloody serum, adhesions between the intestines, and between the intestines and sides of the body. In three instances blood was effused into the peritoneal cavity, in one instance more than a quart; it appeared in this case to come from the liver. The liver was occasionally the seat of this inflammation, not only in its investing membrane, but in the parenchyma; sometimes there were abscesses, and in one instance, portions of it were gangrenous. The lymphatic glands were generally of a dark red colour, frequently resembling clots of blood. This disease of the lymphatic glands was of common occurrence.

The lungs were frequently the seat of this inflammation, portions of one or both presenting different appearances, from simple congestion to complete hepatisation; sometimes there was ulceration, and frequently there was a turbid or sero-purulent, or bloody effusion into the pleural cavity; and often there were extensive adhesions between the lungs and pleura of the ribs. At first, Dr. Sutton was inclined to believe this malady to be a form of pleuro-pneumonia, but his after observations led him to the fact, that the inflammation was not uniformly confined to any organ. In a number of instances, the mucous membrane of the bronchi was deeply inflamed, and the inflammation extended to the trachea and larynx. In several instances the larynx was inflamed, resembling laryngitis. One animal, that had great difficulty in breathing and could make no noise, Dr. Sutton caused to be knocked on the head, and on examination he found the mucous membrane of the larynx and epiglottis inflamed and swollen; also the tongue was swollen. There were evidences in several instances of pericarditis, and produced adhesions between the heart and pericardium. The brain, from the difficulty of opening the skull, was examined only in one instance, it was found healthy, although Dr. Sutton feels confidence in its being frequently the seat of the disease.

In relation to the spread of the disease, all the facts yet ascertained go to prove that it is propagated by contagion. To prove this, Dr. Sutton suggested to the Messrs. Graffs, owners of a distillery at Aurora, to try a series of experimental researches. To this they readily consented; and as they were constantly receiving fresh hogs, there was a fine opportunity to make any experiments that were deemed advisable. The hogs on which the experiments were made, were known to be healthy.

1. Six hogs that had been exposed to the malady, by being in contact with diseased hogs, were put into a yard by themselves and fed on slop and corn; on the fourteenth day from the time they were exposed to the disease, they were all unwell; three died within a week afterwards; the rest recovered.

2. Ninety hogs were exposed to the disease, they were put into a yard by themselves, and fed on corn and water, no slop being given; in thirteen days disease made its appearance among them and they continued to die until sixty out of the ninety died.

3. Fifty hogs were put into a pen by themselves and fed on slop. They had not been exposed to the disease. For six weeks they continued healthy.

4. One hundred hogs that had not been exposed to the disease were put into a yard by themselves, and fed on corn and water; for thirty days no symptoms of disease appeared among them. They were then put into a pen with diseased hogs; on the thirteenth day they began to show symptoms of the malady, and the disease rapidly spread among them until forty died.

5. Thirty-three hogs out of a lot of two hundred and sixty-three, were put into a pen by themselves; for six weeks they continued healthy. The remaining two hundred and thirty were put into a pen adjoining which were diseased hogs. In thirteen days the disease made its appearance among them, and continued until one half died.

6. Four young and healthy hogs were put into a pen, in which, four days previous, diseased hogs had been; they were fed on corn and water. On the fourteenth day they were all unwell, one died on the fifteenth day, and in five days more they were all dead. This experiment shows that infection may be retained in a pen for several days.

7. On the 28th of October, Dr. Sutton inoculated five healthy hogs, from the blood taken from the inflamed tissues of hogs that had died of this disease. On the fourteenth day (November 11th), they were all unwell, and died with but one exception. In three, inflammation spread from the point where they were inoculated, along the skin and down the legs, which became very much swollen. Dr. Sutton does not insist that this inflammation was necessarily caused by the inoculation, as he observes that it did not appear until the fourteenth day, and that many hogs had this external form of disease. From these experiments, Dr. Sutton infers that this disease was infectious, and that the infection had a latent period of from ten to fifteen days. Observations have since led him to consider that the latent period may vary from twelve to twenty days. The experiments also showed, that the hogs in the pens were not dying from strychnine in the slop. A statement was going the round of the papers about this time, that strychnine was used in making yeast in the distilleries, and was poisoning the hogs at these places in large numbers.

The manner in which this disease spread among the hogs from farm to farm in many instances, also showed most conclusively that it was infectious. One farmer had seventy-five hogs turned into a corn-field to fatten. These hogs had been exposed to the disease, had become sickly, and numbers had died. He bought thirty-six more; these were all healthy; they were put into the same field with the diseased hogs; in two weeks they were unwell and numbers died. He bought forty-five more, all healthy, and put them in the same field. In two weeks they began to show symptoms of disease, and in a few days after a number died. Finding that he was likely to lose all his hogs, he sold fifty of the fattest that were left to the butchers in Cincinnati, at a reduced price, as diseased hogs. These hogs, it was said, were purchased for their fat, to be used in the manufacture of lard-oil. After deducting the fifty he sold, he lost out of the one hundred and fifty-six all but ten. A large number of facts could be given, if necessary, to prove the contagiousness of the disease. Hogs that have been once under the influence of this malady appear not to be susceptible to a second attack. Although placed in pens with diseased hogs, they continue healthy and fatten, and not a single instance could there be found of hogs having this disease twice. There is reason to believe that this malady occasionally prevails at the distilleries in a mild form, as the old hogs at many of these places did not take the disease. On the farms, the old and young hogs appeared to be equally susceptible to the disease.

While by these inquiries Dr. Sutton very clearly proved that the disease was directly transmissible from one hog to another, he further endeavoured to ascertain if such transmission were possible to other animals. A dog belonging to Mr. William Ricketts, of Aurora, was chained near the pens at the distillery, and fed on diseased meat; he continued healthy and grew fat, until the sixth week, when he became unwell, vomited a greenish looking mucus, and died on the third day from the time he first showed symptoms of disease. Two more dogs, both belonging to Mr. Woulf Denderline, of Aurora, were also chained near the pens, and fed on diseased meat. One continued healthy until the fifth week, when he refused to eat, vomited a greenish fluid, had diarrhœa, and died on the sixth day from the time he was first attacked. The other dog continued well until the fourth week, when he was taken with vomiting; no diarrhœa was observed, and on the third day after, he died. Two more dogs, belonging to Mr. John Buffington, were chained at the pens, and fed on diseased meat. One of them died in the third week, with similar symptoms to the first mentioned. The other became out of health on the fourth week. The owner thinking he was going to die, had him removed, and fed on different food. He gradually recovered, though it was with difficulty he could walk for more than a month afterwards. I saw him three weeks after he had been removed from the pens. On making him get up, he would stand with his head down, his body drawn up, his back arched, and in attempting to walk would put one foot an inch or so beyond the other. One of the men who attended the hogs, informed Dr. Sutton, that during the sixteen years he had been employed in this manner, he always kept a dog chained near the pens, which he fed on the flesh of the hogs that died, and this food never before injured his dogs.

From the very infectious character of this epizootic, the question may be asked, could this disease be communicated to the human system? Dr. Sutton is of opinion, that there is no evidence that this disease among swine can be communicated to the human subject. The men who attend these animals at the distilleries, have spent nearly their whole time for months together, surrounded by hundreds of diseased hogs, and yet among these men at all the distilleries he could hear from, there was no unusual sickness. Also the soap-boilers and their assistants, who were constantly handling the diseased hogs, and breathing the effluvia arising from their putrefying bodies, often intolerably offensive, do not appear to have been affected. While examining the diseased hogs, Dr. Sutton was several times wounded; but the wounds readily healed, and there was no unusual inflammation.

The possible origin of the disease was another point inquired into by Dr. Sutton; and although his researches were not so satisfactory in this respect, we think it right to give them in full. He found on inquiry that, on June 19th, 1856, one hundred and three hogs were brought to the pens at Aurora, from Ripley County; these hogs were unwell at the time, and some of them died the next day after they were brought in. The hogs in the "pens", up to this time, were healthy. After these diseased hogs had been put into the pens, they continued to die from one to five a day, until about one half died. In two weeks the disease appeared in the adjoining pens, and by the latter part of July, the hogs were dying from about ten to twenty a day. Nearly every hog in these pens, about four thousand, took the disease, and thirty-three per cent died. After the disease appeared in a pen (each pen contains about one hundred hogs), it generally was from six to eight weeks before the hogs that were left recovered. A gentleman who resides at Ripley County, on the second farm from where these hogs were brought, informs me that he lost eighty out of one hundred and fifty; that his neighbour, who lives on the adjoining farm, lost about thirty, and that the disease was prevailing in this neighbourhood before the hogs were taken to Aurora. On these farms, then, in this section of the country, Dr. Sutton considers as one of the places in which the disease originated. He could not ascertain how it was introduced into this neighbourhood. These farms are in the interior of Ripley County, away from the river, not near the railroad, or any other public thoroughfare, nor within twenty miles of a distillery. He thinks that he has evidence also, that it originated in several places in Boon County, Kentucky. That it also, occasionally, originates within the pens at the distilleries, he thinks highly probable. There were four thousand old hogs that continued healthy in the pens at Aurora, while the malady was prevailing, which Dr. Sutton cannot account for, except by considering they might have been under the influence of the disease at some previous period. The introduction of the disease into the distillery in Rising Sun, in Ohio County, was traced to hogs purchased of a farmer in Kentucky. This farmer was sued for damages. A gentleman who was fattening hogs at the distillery, at Patriot, in Switzerland County, states that when he saw the hogs were dying in the county, he refused to buy more, and his hogs continued healthy. Dr. Sutton mentions these facts, because it was stated in the public papers, that this disease originated within the distilleries. He was himself at first inclined to this belief. He thought it was produced from the slop, and from crowding large numbers of hogs together. But he has since fully satisfied himself, that although the disease may, occasionally, arise within the pens at distilleries, it also originates amongst hogs running at large on farms.

From the direct cause of the disease, Dr. Sutton also passes to the consideration of any possible predisposing cause. He relates various meteorological facts, which might be assumed as influencing the disorder; but he shows that these are contradictory, and that evidence is wanting to connect the epizootic with any peculiar atmospheric condition. Altogether, he is clearly opposed to the hypothesis of so-called "epidemic influence".

Such are the facts regarding this new and singular disorder. It reads as a new chapter in disease. The symptoms, as well as the pathology of the disorder, equally point out that the affection belongs to the true inflammatory type. The premonitory depression, the succeeding excitement, the high coloured urine, the buffed blood, the local manifestations, all point to the active inflammatory state, or, in other words, to hyperinosis, as the signal feature of the malady. The disease resembles an acute exanthemata of the human subject, and the more so in that it does not recur. But which of the exanthema? It may have been a form of erysipelas; but if we might offer a suggestion, we should say, that the disease resembles a modified form of scarlet fever, more than any other disorder. It would be an interesting experiment, and one we would respectfully suggest to Dr. Sutton, to inoculate a hog with the blood from a scarlet fever patient, and ascertain whether there could thus be set up a series of symptoms similar to those which Dr. Sutton has so well described.

The idea of the spontaneous origin of the disorder, is one to which we should feel it difficult to subscribe. We should feel a difficulty in such assent in an abstract sense; but the difficulty is still greater when the facts which Dr. Sutton has so laboriously collected are carefully analysed. For while, on the one hand, he offers no proof of spontaneous origin, he gives on the other the best proofs of the necessity of the presence of an infecting animal before the possibility of infection. It is clear, therefore, that the disease has one specific cause: it is feasible that the disease was originally a transplantation to the herd of swine from a human source; or, it is just in the range of possibility, that some organic poison, the product of fermentation at the distilleries, primarily, but capable of reproduction in the organism, was the source of the malady.

We notice in Dr. Sutton's history the omission of one experiment, which should certainly be done. We mean an experiment for ascertaining whether the administration of the liquid excreted matters of a diseased animal, to a healthy one, would propagate the disorder. From the effect of the diseased hog-flesh on the dogs, related in the experiments, we infer that the suggested inquiry would yield an affirmative result. The settlement of this point, would throw considerable light on the mode by which the disease was communicated, and would explain some of the apparent anomalies as to propagation.

We must dismiss from the theory of cause all notion of a meteorological origin for the disorder. The disease is too uniform in all its leading characters, to admit of any other supposition than that, in each and every case, it was the result of a specific poison. Meteorological changes might influence, it is true, the diffusion of the poison. Meteorological changes might influence in some degree the course of the disease in an animal once affected; for in these ways such changes do modify the epidemic influences; but further than this admission, the modern epidemiologist dare scarcely go.

The fact that the disease was not transmissible to the human subject, under the conditions noted by Dr. Sutton, scarcely, as we opine, proves that such transmission was impossible under all circumstances and conditions.

If the disease was, as it would seem to have been, one of our common epidemics in an inferior animal, its retransmission to adult men would, in the majority of cases, be prevented by the circumstance that the people exposed had already been themselves sufferers from the disorder, and therefore were not susceptible, the disease being non-recurrent. From this cause, whole countries of adults escape the ravages of epidemics which are decimating the child population. Nor is it at all surprising, that the flesh of these diseased swine, cooked and eaten, should not communicate the disorder, for it would seem that between cooking and digesting, all mortal organic poisons lose their rancour, and arrive in the blood in new and harmless combinations.

But we must stop. The description of the epizootic fairly supplied, speculation upon it were better held in abeyance. The disease is as yet confined to America, and will, it is to be hoped, find in the Atlantic and Pacific its boundaries. But in a physiological point of view, it is as interesting as the cholera of man, the black death, the sweating sickness, or any other epidemic visitation. We place its history, therefore, before our epidemiologists, as a record of great importance; and in doing so, we beg to offer to Dr. Sutton our respectful and earnest appreciation of his laborious and carefully conducted researches.

---

